# Characterization of human Fab antibody fragments specific to LMP1 (HLEAFab) in nasopharyngeal carcinoma for potential molecular diagnosis and therapeutic applications

**DOI:** 10.3892/ol.2013.1219

**Published:** 2013-02-28

**Authors:** DAWEI ZHANG, YUAN MAO, LIN XIONG, QING CAO, JIN ZHU, RENJIE CHEN

**Affiliations:** 1 Department of Otolaryngology Head and Neck Surgery, The Second Affiliated Hospital of Nanjing Medical University, Nanjing, P.R. China; 2 Department of Otolaryngology Head and Neck Surgery, Jiangsu Provincial Hospital, Nanjing, P.R. China; 3 Department of Pathology, The Second Affiliated Hospital of Nanjing Medical University, Nanjing, P.R. China; 4 Huadong Medical Institute of Biotechniques, Nanjing, P.R. China

**Keywords:** latent membrane protein 1, Fab fragment, BNLF, immunohistochemistry, *in situ* hybridization

## Abstract

In view of the previously demonstrated clinical role of the anti-latent membrane protein 1 (LMP1) immunoconjugate HLEAFab-MMC in the treatment of advanced nasopharyngeal carcinoma (NPC), reliable detection of LMP1 expression is of key importance. The aim of this study was to investigate LMP1 status in NPC. Tissue samples from 36 cases were analyzed by immunohistochemistry (IHC) and *in situ* hybridization for LMP1 and BNLF1 (LMP1 gene) expression, respectively. The results showed LMP1 expression in 20/36 (55.6%) cases in the HLEAFab group compared with 22/36 (61.1%) cases in the S12 group (purified mouse anti-human latent membrane protein 1 monoclonal antibody). The positive staining for LMP1 in the tumor cell membranes exhibited uniformity between HLEAFab and S12. BNLF1 was observed to be amplified in 19/36 NPC patients (52.8%). The sensitivities of HLEAFab-IHC-positivity and S12-IHC-positivity for amplification were 84.2 and 89.5%, respectively. Positive expression of LMP1 was present in a significant proportion of the NPC samples. The present findings provide an important strategy for molecular therapy targeting LMP1 in NPC patients.

## Introduction

Nasopharyngeal carcinoma (NPC) is particularly common in areas of China and South-East Asia, and is highly associated with Epstein-Barr virus (EBV) infection (1). EBV infection is present in all poorly and undifferentiated non-keratinizing NPC regardless of geographic origin, and the viral antigens expressed by NPC provide potential targets for immunotherapy.

HLEAFab is a human monoclonal antibody directed against latent membrane protein 1 (LMP1), which is considered to be the most important oncoprotein encoded by EBV. We have previously investigated the effects of an immunoconjugate (HLEAFab-MMC) on proliferation and apoptosis in the NPC HNE2/LMP1 cell lines, as well as the inhibition of the growth of NPC xenografts in nude mice (1). The expression of LMP1 varies in various types of NPC. LMP1 is expressed and detectable in only 20–65% of NPC patients, using western blotting, immunohistochemistry (IHC) or other assays (2,3). Therefore, the biological efficacy of the Fab-based immunoconjugate varies in EBV-related carcinomas with various levels of LMP1 expression. Patients with LMP1-positive cancers were shown to benefit from the effect of the immunoconjugate on standard chemotherapy.

Prior to initiation of the targeted therapy with HLEAFab-MMC, the expression of LMP1 was evaluated with IHC and *in situ* hybridization (ISH) in samples from 36 NPC patients. The present study shows that the HLEAFab antibody fragment has an excellent correlation with S12 in IHC-positive cases and ISH also yields identical results. S12 is a purified mouse anti-human latent membrane protein 1 monoclonal antibody from, which is used as the standard control as it has in other studies (4). HLEAFab may be an ideal candidate for NPC treatment using the novel immunoconjugate of HLEAFab-MMC.

## Patients and methods

### Patients

A total of 36 cases of NPC were selected from the Department of Otolaryngology Head and Neck Surgery, Second Affiliated Hospital of Nanjing Medical University (Nanjing, China) between 2002 and 2010. The diagnosis of NPC was determined according to the latest WHO criteria (5). The study was approved by the Ethics Committee of The Second Affiliated Hospital of Nanjing Medical University, Nanjing, China. Written informed consent was obtained from the patient for publication of this study and any accompanying images.

### IHC for LMP1 expression

The EnVision method was used for immunohistochemical analysis (6–8). NPC tissues were excised and sent for paraffin wax-embedding and processing. Sections (5-*μ*m thick) were deparaffinized with xylene, then dehydrated in decreasing concentrations of alcohol. Endogenous peroxidase activity was blocked by hydrogen peroxidase (3%) in Tris-buffered saline (TBS) for 30 min. The sections were then boiled for 10 min under pressure in citrate buffer for antigen retrieval. Nonspecific binding was blocked with 5% goat serum in TBS for 15 min and the tissues were incubated with HLEAFab and S12 antibodies (cat. 559898; Becton Dickinson Medical Devices Co. Ltd, Franklin Lakes, NJ, USA) in TBS containing 1% bovine serum albumin for 60 min. The sections were then washed with TBS and incubated with goat Anti-Human IgG (Fab specific)-peroxidase (Sigma, St. Louis, MO, USA; Cat. No. A0293; 1:100) and EnVision goat anti-mouse IgG peroxidase antibody (EB-2305, ZhongShan, Godbridge, China; 1:200) for 60 min. The color was developed in diaminobenzidine solution and counterstained with Mayer’s hematoxylin.

### Preparation of DIG-labeled probes and ISH

LMP1 probes were prepared under the following conditions: i) DNA was extracted from a 3-mm thick section based on published protocols for formalin-fixed, paraffin-embedded tissues (9). Sections were digested with proteinase K (Roche Molecular Biochemicals, Mannheim, Germany). The samples were then centrifuged to pellet the cell debris and the supernatant was used for PCR amplification. The PCR template was performed with primers 1 and 2 (primer 1, 5′-AGAAACACGCGTTACT CTGACG-3′; primer 2, 5′-ACAATGCCTGTCCGTGCAAAT TCC-3′) using a PCR instrument (MJ Research Inc., St. Bruno, QC, Canada). Amplification was performed with 30 cycles at 90°C for 1 min, 55°C for 1 min and 72°C for 1 min and the final extension was at 72°C for 15 min. The PCR product, which was the first exon of the LMP1 gene (BNLF1), was analyzed on an ethidium bromide-stained 1.5% agarose gel. The positive control for the PCR reactions contained the fragment of the plasmid DNA BNLF1 in double distilled water for disinfection (10). ii) Digoxin-labeled probe: The PCR-positive product was used as a template for PCR DIG Labeling Mix (Roche Molecular Biochemicals; Cat. No. 11585550910) and primers 1 and 2 were synthesized by PCR probes according to product instructions, followed by ultrafiltration unit (Millipore) centrifugation to remove free dNTP and the purified probe. iii) ISH was performed with the LMP1 gene in all cases. Sections (5-*μ*m) were deparaffinized prior to the RNA enzyme treatment to remove RNA and then placed in pretreatment solution at 95°C for 10 min, followed by 15 min cooling at room temperature. Sections were then washed twice with wash buffer for 3 min. After being blocked with 3% H_2_O_2_ and methanol for 30 min and digested by proteinase K for 20 min with thorough rinsing, the sections were post-fixed in 4% cold PFA-PBS for 15 min. The sections were then rinsed with PBS and distilled water, pre-hybridized for 4 h and hybridized with 100 *μ*l of digoxigenin-labelled LMP1 probe for 18 h at 42°C. Following thorough rinsing with SSC and incubation in blocking solution, the slides were processed with mouse anti-digoxin-biotin antibody, SABC-POD and streptavidin-HRP at 37°C, in sequence. At each step, the slides were rinsed three times with PBS. Finally, the slides were colored with DAB and counterstained with hematoxylin (11).

### Statistical analysis

SPSS 18.0 (SPSS, Inc.) was used for all statistical analyses. The Pearson χ^2^ test was used to compare discrete variables. P≤0.05 was considered to indicate statistically significant differences for all comparisons.

## Results

### Tumor characteristics

The 36 NPC samples were subjected to IHC and ISH analyses. Among the cases, there were 16 females and 20 males. The mean age was 61.58 years (range, 42–78 years). Of the 36 cases, 2 were squamous cell carcinoma (5.5%), 11 were non-keratinizing tumors (30.6%) and the remaining 23 cases (64%) were undifferentiated.

### IHC

**Figure 1:**
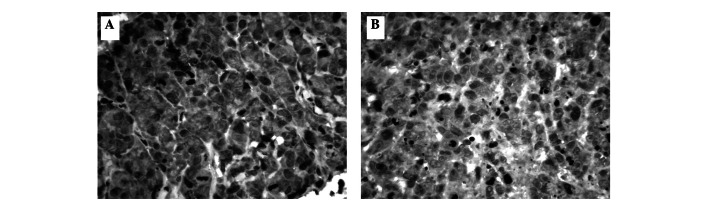
Formalin-fixed, paraffin wax-embedded tissue sections of NPC tissue with cell membranes stained using (A) HLEAFab or (B) S12. Positive staining was observed in the NPC cell membrane and cytoplasm. Visualization using DAB/H_2_O_2_ (light gray). Magnification, ×200. NPC, nasopharyngeal carcinoma.

Positive staining with HLEAFab or S12 was observed in the NPC cell membranes and cytoplasm (Fig. 1). LMP1 expression was observed in 20/36 (55.6%) cases in the HLEAFab group and 22/36 (61.1%) cases in the S12 group. Similarly, LMP1 expression was detected in 2 squamous cell carcinoma cases, 7/11 cases of non-keratinizing tumors and 13/23 cases of undifferentiated tumors in the HLEAFab group, compared with the same 2 squamous cell carcinoma cases, 6/11 cases of non-keratinizing tumors and 16/23 cases of undifferentiated tumors in the S12 group (Table I). Positive staining for LMP1 was not significantly different between the HLEAFab and S12 groups (P>0.05). Staining was entirely absent with the unrelated antibody Fab fragment as the negative control antibody.

### ISH

**Figure 2:**
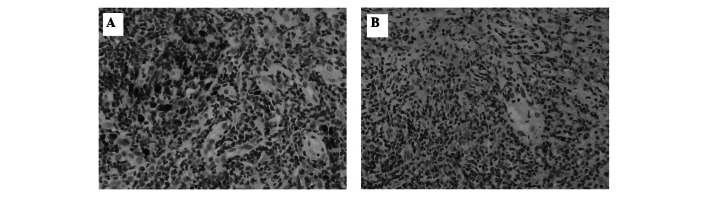
*In situ* hybridization using (A) positive or (B) negative LMP1 probes (visualization of NPC tissue cell nuclei, black). Magnification, ×100. LMP1, latent membrane protein; NPC, nasopharyngeal carcinoma.

The *in situ* detection of BNLF1 using an LMP1 probe in NPC sections is shown in Fig. 2. BNLF1 was observed in 19/36 NPC cases (52.8%). Among all the positive cases, 16/20 (80%) were amplified with positive IHC staining in the HLEAFab group, while 3/16 (19%) were amplified with negative IHC staining. By comparison, 17/22 (77%) cases were amplified with positive IHC staining in the S12 group, while 2/14 (14%) were amplified with negative IHC staining. There was only 1 case with negative IHC staining in the HLEAFab group but positive IHC staining in the S12 group (Table II). The sensitivity of the amplification was 84.2% for the IHC-positive cases in the HLEAFab group and 89.5% in the S12 group. The specificity was 76.5% for the IHC-positive cases in the HLEAFab group and 70.6% in the S12 group. The ISH results of the 36 samples were consistent with the IHC data (P>0.05).

## Discussion

LMP1, which is encoded by EBV, is significantly expressed in several EBV-associated malignancies, particularly NPC. LMP1 is an oncoprotein which acts by constitutive activation of various signaling pathways and induces the promotion of cell growth and inhibition of apoptosis in a variety of cell types *in vitro*(12). In addition, LMP1 has been demonstrated to contribute to B cell and epithelial cell tumourigenesis *in vivo* in 4 transgenic mice (13–15). These unique characteristics of LMP1 lead to the suggestion that LMP1 may be a good therapeutic target in the treatment of EBV-associated carcinomas. Numerous studies have shown that certain antibodies are able to target LMP1 successfully by binding to its intracellular activating regions (16–18). In our previous study, HLEAFab was a humanized antibody fragment Fab directed against LMP1, targeting the extramembrane domains (EMD) of the polypeptide.

In the present study, IHC was used for detection of LMP1 with HLEAFab and S12 antibodies. Positive staining of LMP1 was observed in the membrane and cytoplasm of NPC cells. LMP1 staining was detected in 55.6% cases in the HLEAFab group compared with 61.1% cases in the S12 group. LMP1 staining was not significantly different between the HLEAFab and S12 groups (P>0.05). BNLF1 was observed in 19/36 NPC cases (52.8%). The sensitivity for amplification was 84.2% for the HLEAFab group and 89.5% for the S12 group with positive IHC cases. The specificity was 76.5% for HLEAFab-positive IHC and 70.6% for S12-positive IHC. The results showed that the antibody fragment HLEAFab had an excellent correlation with S12 in IHC-positive cases and ISH analysis yielded identical results in 36 samples.

This poor immunogenicity, particularly due to the short extracellular structure of LMP1, may explain why IHC exhibited different results for EBV-LMP1 with regard to the reactivity of HLEAFab and S12. HLEAFab, a humanized anti-LMP1 EMD Fab fragment, is able to bind to the extra-cellular domains of LMP1. LMP-specific mAb S12 and other monoclonal antibodies are known to recognize large intracellular regions of LMP1 for ligand binding. Consequently, LMP1 expression in the present study was shown to be identical when using HLEAFab or S12 in the IHC method.

The lack of detectable BNLF amplification in 4 cases of IHC-positive staining in the HLEAFab group and 5 cases in the S12 group, as well as BNLF amplification in 3 cases of negative staining in the HLEAFab group and 3 cases in the S12 group, indicates that LMP1 expression is not required for the maintenance or regulation of latent EBV infection in epithelial cells and thus BNLF1 fragments may be lost following EBV-induced cell transformation in the cell differentiation and proliferation process. Modulation of LMP1 expression is affected by a number of regulatory gene factors, as well as the heterogeneity of host cells (19,20). These lead to the positive expression of BNLF1 fragment without LMP1 expression in certain cases, as observed in the present study.

The search for targeted therapies for NPC has been continuing. As the number of studies is growing, standardization of all steps of the testing process from tissue fixation to final interpretation is likely to ensure accurate identification of patients who may benefit from specific therapies. Preclinical trials with the antibody Fab fragments are ongoing to broaden the spectrum of possibilities of targeted approaches to the personalized treatment of NPC.

## Figures and Tables

**Table I:** Comparison of LMP1 expression by IHC with HLEAFab and S12.

	HLEAFab			S12		
	Negative	Positive	Total	Negative	Positive	Total
Squamous cell carcinoma	2	0	2	2	0	2
Non-keratinizing	4	7	11	5	6	11
Undifferentiated	10	13	23	7	16	23
Total	16	20	36	14	22	36

LMP1, latent membrane protein 1; IHC, immunohistochemistry.

**Table II:** Comparison of LMP1 expression by ISH with HLEAFab and S12.

	Negative	Positive	Total
HLEAFab (−)	13 (81%)	3 (19%)	16
HLEAFab (+)	4 (20%)	16 (80%)	20
S12 (−)	12 (86%)	2 (14%)	14
S12 (+)	5 (23%)	17 (77%)	22
Total	17	19	36

LMP1, latent membrane protein 1; ISH, *in situ* hybridization.

## References

[1] Chen R, Zhang D, Mao Y, Zhu J, Ming H, Wen J, Ma J, Cao Q, Lin H, Tang Q, Liang J, Feng Z (2012). A human Fab-based immunoconjugate specific for the LMP1 extracellular domain inhibits nasopharyngeal carcinoma growth *in vitro* and *in vivo*. Mol Cancer Ther.

[2] Young LS, Dawson CW, Clark D, Rupani H, Busson P, Tursz T, Johnson A, Rickinson AB (1988). Epstein-Barr virus gene expression in nasopharyngeal carcinoma. J Gen Virol.

[3] Brooks L, Yao QY, Rickinson AB, Young LS (1992). Epstein-Barr virus latent gene transcription in nasopharyngeal carcinoma cells: coexpression of EBNA1, LMP1, and LMP2 transcripts. J Virol.

[4] Jiwa NM, Oudejans JJ, Dukers DF, Vos W, Horstman A, van der Valk P, Middledorp JM, Walboomers JM, Meijer CJ (1995). Immunohistochemical demonstration of different latent membrane protein-1 epitopes of Epstein-Barr virus in lymphoproliferative diseases. J Clin Pathol.

[5] Barnes L, Eveson JW, Reichart P, Sidransky D, World Health Organization Classification of Tumours (2005). Pathology and Genetics of Head and Neck Tumours.

[6] Mao Y, Zhang DW, Wen J, Cao Q, Chen RJ, Zhu J, Feng ZQ (2012). A novel LMP1 antibody synergizes with mitomycin C to inhibit nasopharyngeal carcinoma growth *in vivo* through inducing apoptosis and downregulating vascular endothelial growth factor. Int J Mol Sci.

[7] Mao Y, Zhang DW, Zhu H, Lin H, Xiong L, Cao Q, Liu Y, Li QD, Xu JR, Xu LF, Chen RJ (2012). LMP1 and LMP2A are potential prognostic markers of extranodal NK/T-cell lymphoma, nasal type (ENKTL). Diagn Pathol.

[8] Mao Y, Zhang DW, Lin H, Xiong L, Liu Y, Li QD, Ma J, Cao Q, Chen RJ, Zhu J, Feng ZQ (2012). Alpha B-crystallin is a new prognostic marker for laryngeal squamous cell carcinoma. J Exp Clin Cancer Res.

[9] Pan LX, Diss TC, Peng HZ, Isaacson PG (1994). Clonality analysis of defined B-cell populations in archival tissue sections using microdissection and the polymerase chain reaction. Histopathology.

[10] Zeng W, Zhou M, Lin H (1997). The significance of detecting Epstein-Barr virus BNLF1 fragment and its expression in Hodgkin’s disease in the Guangdong area. Zhonghua Bing Li Xue Za Zhi.

[11] QingLing Z, LiNa Y, Li L, Shuang W, YuFang Y, Yi D, Divakaran J, Xin L, YanQing D (2011). LMP1 antagonizes WNT/β-catenin signalling through inhibition of WTX and promotes nasopharyngeal dysplasia but not tumourigenesis in LMP1 (B95-8) transgenic mice. J Pathol.

[12] Kieff E, Rickinson AB, Knipe M, Howley PM (2001). Epstein-Barr virus and its replication. Field’s Virology.

[13] Wilson JB, Weinberg W, Johnson R, Yuspa S, Levine AJ (1990). Expression of the BNLF-1 oncogene of Epstein-Barr virus in the skin of transgenic mice induces hyperplasia and aberrant expression of keratin 6. Cell.

[14] Kulwichit W, Edwards RH, Davenport EM, Baskar JF, Godfrey V, Raab-Traub N (1998). Expression of the Epstein-Barr virus latent membrane protein 1 induces B cell lymphoma in transgenic mice. Proc Natl Acad Sci USA.

[15] Stevenson D, Charalambous C, Wilson JB (2005). Epstein-Barr virus latent membrane protein 1 (CAO) up-regulates VEGF and TGF alpha concomitant with hyperlasia, with subsequent up-regulation of p16 and MMP9. Cancer Res.

[16] Gennari F, Mehta S, Wang Y, St Clair Tallarico A, Palu G, Marasco WA (2004). Direct phage to intrabody screening (DPIS): demonstration by isolation of cytosolic intrabodies against the TES1 site of Epstein Barr virus latent membrane protein 1 (LMP1) that block NF-kappaB transactivation. J Mol Biol.

[17] Fang CY, Chang YS, Chow KP, Yu JS, Chang HY (2004). Construction and characterization of monoclonal antibodies specific to Epstein-Barr virus latent membrane protein 1. J Immunol Methods.

[18] Piché A, Kasono K, Johanning F, Curiel TJ, Curiel DT (1998). Phenotypic knock-out of the latent membrane protein 1 of Epstein-Barr virus by an intracellular single-chain antibody. Gene Ther.

[19] Salamon D, Adori M, Ujvari D, Wu L, Kis LL, Madapura HS, Nagy N, Klein G, Klein E (2012). Latency type-dependent modulation of Epstein-Barr virus-encoded latent membrane protein 1 expression by type I interferons in B cells. J Virol.

[20] Ning S, Hahn AM, Huye LE, Pagano JS (2003). Interferon regulatory factor 7 regulates expression of Epstein-Barr virus latent membrane protein 1: a regulatory circuit. J Virol.

